# Bridging theory and data: A computational workflow for cultural evolution

**DOI:** 10.1073/pnas.2322887121

**Published:** 2024-11-18

**Authors:** Dominik Deffner, Natalia Fedorova, Jeffrey Andrews, Richard McElreath

**Affiliations:** ^a^Center for Adaptive Rationality, Max Planck Institute for Human Development, 14195 Berlin, Germany; ^b^Science of Intelligence Excellence Cluster, Technical University Berlin, 10623 Berlin, Germany; ^c^Department of Human Behavior, Ecology and Culture, Max Planck Institute for Evolutionary Anthropology, 04103 Leipzig, Germany

**Keywords:** cultural evolution, workflows, causal inference, anthropology, computational modeling

## Abstract

Cultural evolution applies evolutionary concepts and tools to explain the change of culture over time. Despite advances in both theoretical and empirical methods, the connections between cultural evolutionary theory and evidence are often vague, limiting progress. Theoretical models influence empirical research but rarely guide data collection and analysis in logical and transparent ways. Theoretical models themselves are often too abstract to apply to specific empirical contexts and guide statistical inference. To help bridge this gap, we outline a quality-assurance computational workflow that starts from generative models of empirical phenomena and logically connects statistical estimates to both theory and real-world explanatory goals. We emphasize and demonstrate validation of the workflow using synthetic data. Using the interplay between conformity, migration, and cultural diversity as a case study, we present coded and repeatable examples of directed acyclic graphs, tailored agent-based simulations, a probabilistic transmission model for longitudinal data, and an approximate Bayesian computation model for cross-sectional data. We discuss the assumptions, opportunities, and pitfalls of different approaches to generative modeling and show how each can be used to improve data analysis depending on the structure of available data and the depth of theoretical understanding. Throughout, we highlight the significance of ethnography and of collecting basic cultural and demographic information about study populations and call for more emphasis on logical and theory-driven workflows as part of science reform.

## Significance

Cultural evolution has made remarkable progress in both our theoretical and empirical understanding of how culture changes through time. However, a crucial challenge persists: closing the gap between theoretical models and real-world data. We propose a computational workflow that starts with theoretical models and directly guides data analysis, focusing on logically and transparently connecting theoretical implications with statistical procedures. Importantly, we also show how to step back from the basic outputs of data analysis to implications for both the studied population and theoretical model, highlighting avenues for the use of both ethnographic and experimental information. We argue these steps are not only necessary for principled research but contribute key insights to recent debates on scientific reform.

Half a century ago, the study of cultural evolution was reinvigorated with formal theory, adapting concepts and tools from population biology to describe the mechanisms governing cultural change (1–4). As a consequence of its history, the field of cultural evolution is characterized by a shared and growing body of quantitative theory. Using formal models, researchers have uncovered the conditions favoring the evolution of social learning and of specific social learning strategies (or “transmission biases”), and have identified how individual-level cognitive and demographic processes generate cultural change at the population level (see refs. 5 and 6 for reviews). The field’s theoretical origins fostered a rapidly growing empirical literature. Controlled experiments and observation of cultural transmission in natural populations have become integral parts of cultural evolution research, highlighting both the pervasive role that culture plays for humans and other animals as well as the breadth of the field of cultural evolution.

For all this success, the links between theory and empirical data analysis are often weak, hindering further progress and integration. Empirical research is often inspired by modeling work but does not directly use this theory to design data collection or to analyze the obtained data. Theoretical models, in turn, are often motivated by empirical case studies, but are typically constructed at a level of abstraction that makes them impossible to apply directly to any particular real-world system. To give an example, theory addresses the drivers of cumulative culture over orders of magnitude of population size and intergenerational timescales (7–10). Experiments claiming to test the theories of cumulative culture describe instead convenient and noisy measures of task improvement in small groups over few rounds of transmission (11, 12). Observational studies sometimes have the same problems, but without any experimental control. Big claims of theoretical relevance, without any formal predictions nor logic connecting theory to measurements, are easy to find in highly cited cultural evolution papers (13–16). These studies do not use cultural transmission models to analyze the data, relying instead on linear models, group comparisons, and prediction, without a clear logic of causal identification. When the links between theory, measurement, and analysis are metaphorical and rhetorical, there can be little scientific progress. Of course, other fields have similar problems with bridging theory and empiricism, but this article is not about those fields.

In the rest of this article, we outline a computational workflow for bridging theory and data in cultural evolution research. We describe how generative cultural evolutionary models allow researchers to logically connect abstract theory to the real world and inform data-analytic procedures. Generative models embody causal assumptions about the phenomena of interest and are, thus, capable of simulating predictions. We describe different forms such models can take, ranging from directed acyclic graphs (DAGs) to agent-based simulation models (ABMs). We compare their respective assumptions and fields of application and outline how each approach can be used to improve data analysis. Similar workflows involving model validation with synthetic experimental data and posterior simulations are already rather commonplace in the fields of cognitive modeling and computational psychology (17–20). Generative computational workflows have also been employed by cultural evolution researchers, mostly analyzing behavior within social learning experiments (21–25); but such workflows still need to become more routinely used and also better connected to abstract theory for both experimental and observational scenarios. To facilitate the adoption of this generative approach, we illustrate a complete workflow using the interplay between (anti)conformist transmission, migration, and cultural diversity as a case study (3, 26, 27). We showcase how to move between theory and data providing 1) high-level generative models, 2) tailored agent-based models, 3) probabilistic transmission models for time-series data, and 4) approximate Bayesian computation (ABC) models for cross-sectional data, all including annotated code in our online repository (28). In real projects, these components scaffold and constrain one another, with insights moving in all directions.

## Toward a Principled and Validated Workflow

**Fig. 1.**
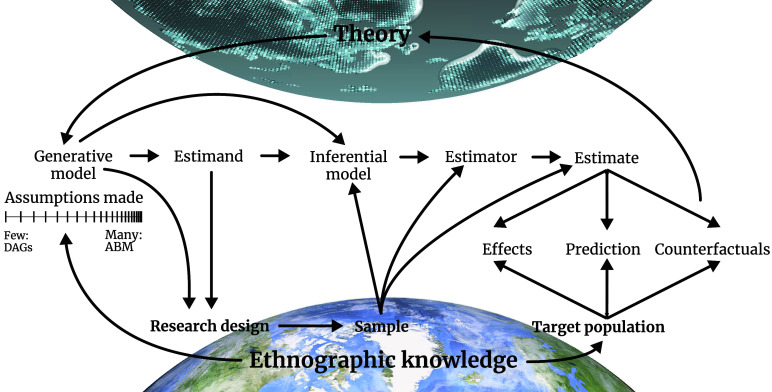
Illustration of a computational workflow for cultural evolution that connects abstract theory to real-world data. The arrows indicate logical and computational dependencies among theoretical, statistical, and scientific elements of a project. For example, an effective inferential model depends upon generative models, a question about those models (estimand), and the structure of the available evidence (sample).

Cultural evolution is arguably in a privileged position among the social sciences in that it has an extensive catalog of formal modeling to build upon (29, 30). But operationalizing this work in the analysis of real-world data is not trivial. We posit that connecting theory to data requires a made-to-measure computational workflow (31), starting from generative models of the phenomenon at hand and ending with statistical estimates that are logically connected to our inferential goal and target scenario. Fig. 1 shows an illustration of the workflow; we first describe the components in abstract terms before turning to a concrete example.

### Generative Models.

Generative models represent causal assumptions about the (latent) processes generating the observed data or, more formally, joint probability distributions over all parameters and data [note that the distinction between generative and nongenerative models is not binary (31)]. Generative models are concrete instantiations of theory, varying in their explanatory detail. And they provide synthetic data that can be used to validate later statistical and computational choices.

DAGs can be considered the simplest form of generative models. They are static representations of dynamic systems at equilibrium and abstractly describe how variables change as a function of other variables (32–36). This lack of specificity can be a strength, because the implications we deduce from DAGs consequently do not depend on detailed assumptions, and communication of important structural assumptions is easy with details omitted.

Still, cultural evolutionary theory is inherently dynamic and to make data speak to such theory, more dynamic generative models are needed that explicitly link individual-level processes to cultural dynamics over time. Dynamic models can either explicitly model individuals, like agent-based simulations (30), or they can describe aggregated population-level quantities, like classic population genetic-type cultural evolution models (1–3); they can unfold in discrete or continuous time, and they can follow deterministic or stochastic algorithms (30, 37). In general, more mechanistic models provide a richer understanding of a specific phenomenon but may also come at a cost of lower generality and greater potential for misspecification.

No one-size-fits-all solution exists and the appropriate detail of a generative model depends on the explanatory goal and the state of our theoretical understanding. Studying the same phenomena at different levels of abstraction provides its own benefits. Working at different scales, generative models give researchers the space to explore the consequences of information they have on the phenomena under study and, thereby, help translating between abstract theories and real-world study systems.

### From Estimand to Estimate.

Many papers connect theory and data by means of story-telling; story-telling does not formally define a target of inference (i.e., an estimand) and bases its conclusions on statistical estimators that are only metaphorically related to theoretical constructs. The starting point for any empirical analysis that aims to logically connect theory and data is to define the estimand within the context of the generative model. The estimand is the specific object of inference, representing the goal of our analysis and the quantity we aim to estimate (see ref. 38, for an introduction). For example, the estimand might be the effect of migration on cultural diversity for a given level of conformity in our study population, with formal definitions of diversity and conformity in a specific demographic context. Once determined, we can select appropriate control variables for inferential (statistical) models and determine identifiability, i.e., whether the effect of interest can be estimated at all with the data at hand (33, 39). The estimand creates a direct, logical connection between the generative and inferential models and typically also includes the target population, providing a link back to the real world. Being specific about the estimand tells us which variables are necessary, and which must be ignored, for proper identification.

Using the generative model to simulate synthetic data is essential to understanding the inferential limits of a potential dataset. A nightmare scenario is to invest considerable effort and funds into collecting data that have no hope of answering the research question, as data can often be too sparse and noisy. When studying evolutionary processes, equifinality is also a primary concern. Equifinality means that, even in the absence of classical confounding, many competing processes can be compatible with the same empirical pattern, raising questions about inferential power (40, 41). Finally, the huge diversity in human lifeways means that cultural evolutionary processes are never isolated in empirical systems and this makes generalization and comparison dependent upon generative assumptions (42, 43). For example, in a population with limited migration, conformity might be a strong driving force, while in another population, high levels of migration might wipe out the effect. Simulation methods can help us “stress-test” analytic procedures, allowing us to conduct in silico experiments to explore whether the cultural evolutionary dynamic can be studied in our population of interest and whether our analysis is sensitive enough to capture differences in parameter values.

Once the empirical sample is collected, we can set up an inferential model and use one or more statistical estimators (e.g., a Bayesian network) and algorithms (optimization, MCMC, ABC) to derive estimates for our target of analysis. Much has been written about the derivation and construction of estimators and target estimates. This is a general topic in scientific computation that work in cultural evolution can benefit from (see, e.g., refs. 36, 44, and 45 for popular advanced textbooks on Bayesian inference that take the generative perspective).

### Causal Effects, Predictions, and Counterfactuals.

Statistical estimators provide us with model coefficients, but reporting and interpreting raw statistical estimates including their associated uncertainty intervals, *P*-values, or Bayes factors is only an intermediate step in empirical analyses. Parameter estimates themselves are hard to interpret (especially in non-Gaussian models and models with interaction terms) and sizable differences on the parameter (e.g., logit) scale may correspond to negligible changes on the outcome (e.g., probability) scale. Therefore, answering research questions in the context of the target population often requires additional postprocessing of model estimates. First, to compute causal effects, we need to project our estimates to the outcome scale and to the relevant target population and potentially average (or “marginalize”) over other relevant variables jointly causing the outcome (46, 47). Second, using the assumptions embodied in the generative model in combination with data from a separate target population, we can compute predictions or generalizations beyond the study sample (43). As comparisons between populations are implicit exercises in generalization as well, this procedure also allows researchers to compare societies on an equal footing. Third, using the generative model, researchers can make principled counterfactual inferences about “what would have been” under different cultural or demographic circumstances. For example, we could estimate how cultural diversity in a specific population would change if the level of conformity would have been slightly higher than it is in reality.

Finally, researchers can now loop back to theory and iteratively update the generative model based on comparisons between computed effects, model predictions, and posterior simulations. Insights about inferential limits and potentials can inform future research design and data collection and, following many iterations of this process, improve our understanding of the processes generating the observed cultural dynamics.

## An Example Workflow: Migration and Conformity

To substantiate our argument and provide resources for others to adopt this generative approach, we now work through a comprehensive example, analyzing how conformity and migration jointly shape cultural diversity. We demonstrate how to formalize generative models through DAGs and agent-based simulations, how to define estimands and translate them into statistical models and how to directly fit a dynamical generative model to longitudinal and cross-sectional data. The specific techniques in this example are broadly useful, but the core requirement is to adopt a formal workflow that makes transparent and computational justifications for each step. While we focus on an observational case study, the general computational workflow is equally suited for experiments. Indeed, the greater control of experimental situations typically makes the formulation, fitting, and testing of precise generative models more straightforward (18, 20). Likewise, we do not outline a strictly linear workflow. Rather, we provide different flavors of generative and inferential models that researchers may consider depending on the assumptions they are willing to make and the data they have available.

### Real-World Phenomenon.

Many groups grapple with the question: what will come of their cultural traditions amid the influence of migration? Migration brings new people and diverse cultures and, thus, has the potential to change a group’s cultural fabric. On one end of the spectrum, migrants may face substantial pressure to assimilate into local practices, preserving the existing cultural landscape. On the opposite end, they might retain their original cultures, successfully disseminating the beliefs and values of their homelands, thereby creating a novel cultural mosaic. Given varying migration rates and social pressures to conform to majority behavior, how would we expect these cultural evolutionary dynamics to play out?

### Theory.

Understanding such processes has been a key goal of theory in cultural evolution, but the study of how culture changes has much deeper origins. To give just one example, applying his theory of “cultural ecology,” anthropologist Julian Steward described cultural change in the Great Basin as a result of environmental adaptations, migration, and social interactions (48, 49), paving the way for formalization in a cultural evolutionary framework. In their original modeling work, Boyd and Richerson have shown that spatially varying environments can favor the evolution of “conformist” transmission, i.e., social learning where frequent variants are disproportionately more likely to be copied; they further showed that such conformity can maintain similarities within and differences between cultural groups in the face of migration that would otherwise erode group differences (3). Henrich and Boyd (26) and Nakahashi (50) extended this work to temporally as well as spatially varying environments and revealed that conformity is adaptive across a much wider range of conditions, suggesting that it might be widespread in natural populations. Finally, bringing those abstract models closer to the real world, Mesoudi (27) linked conformity and realistic migration rates to cultural $F_{\mathrm{ST}}$, a quantitative measure of between-group cultural structure that has frequently been used in the empirical literature (51–54). The aforementioned studies show the advance in theory, providing a cumulative corpus that would benefit from an empirical test. How would one go about testing this theory with empirical data from a contemporary population?

### Directed Acyclic Graphs (DAGs).

A widespread way of logically and formally connecting theory to data is through DAGs. Multiple comprehensive yet accessible introductions to DAGs are available (32–36, 55), and reading one or more is necessary to appreciate and understand the approach. DAGs represent abstract generative models. In DAGs nodes represent variables and arrows represent causal effects. Using DAGs we can express assumptions about which variables change if we intervene on a set of other variables. And this allows us in turn to logically derive statistical procedures from specific questions about causal effects. Using DAGs we can 1) identify testable implications, 2) deduce adjustment sets, 3) compute generalizations, and 4) simulate empirical expectations. Models in cultural evolution can often be expressed at different levels of organization, from individual to population (below we show how dynamical models can explicitly link multiple levels over time). Here, our DAGs represent population-level models where populations comprise multiple subgroups with migration occurring between them.

**Fig. 2.**
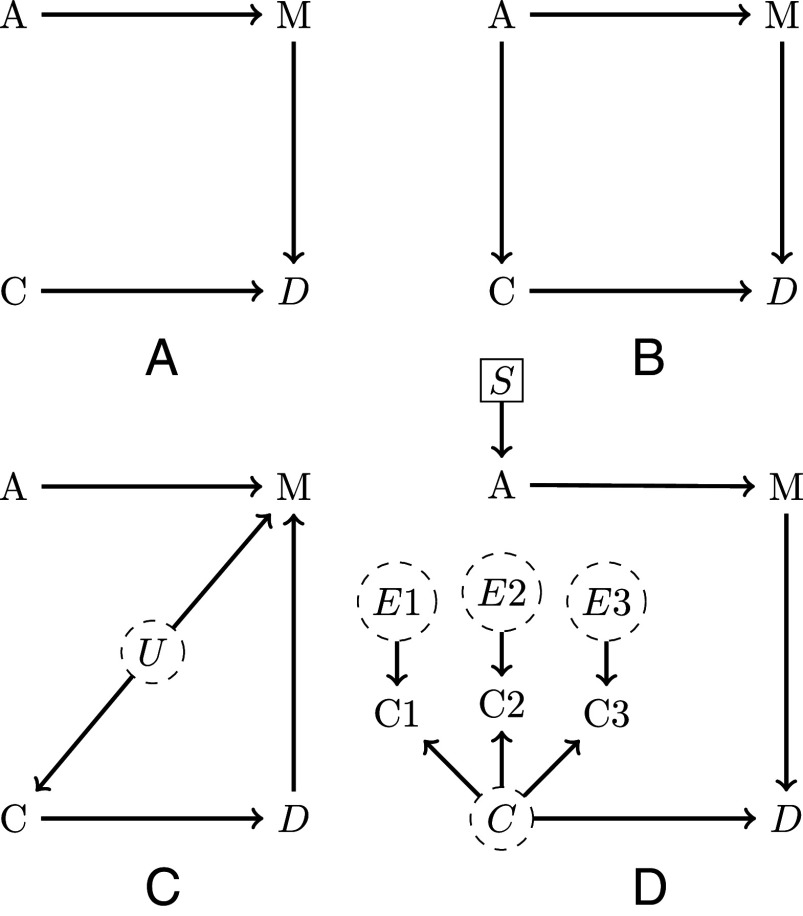
DAGs representing assumptions about the causal effects between age *A*, conformity *C*, migration rate *M*, and cultural diversity *D* (panels *A*–*D* represent different sets of assumptions; see text for detailed explanations). Dashed circles denote unobserved variables and selection nodes S indicate mechanisms by which populations might differ.

The DAG in Fig. 2*A* says that the structuring of cultural diversity (*D*) in a population changes as a function of conformity (*C*) and migration (*M*), which, in turn, depends on age (*A*; e.g., young people might be more likely to migrate leading to more migration among subgroups in populations with a younger age structure). But the diagram does not assume any form (e.g., linear) for these relationships nor the interactions between variables (e.g., how do *C* and *M* interact in causing *D*). In Fig. 2*A*, both migration and conformity affect cultural diversity through distinct paths. From this, we learn that to assess the impact of conformity on cultural diversity, researchers need not “control” for any other variable [there are no open “backdoor paths” (33, 36)]. However, we can see that the model makes a key assumption, that migration and conformity rates are causally independent. Below, we discuss how such “testable implications” can be used to assess the compatibility of DAGs with empirical evidence.

Fig. 2*B* introduces a single modification. Here, age not only influences migration but also conformity. In such cases, conditioning on [stratifying by (56)] *M* is necessary for an accurate estimate of C→D. A key virtue of deriving statistical procedures from generative models, DAGs or otherwise, is that some variables can actually damage inference. It is not harmless to just add everything and let statistical diagnostics sort it out. Fig. 2*C* depicts a scenario where controlling for a variable may be detrimental (39). Migration rate (*M*) here is influenced by age (*A*), diversity (*D*), and unobserved cultural variables (*U*; e.g., family, ingroup-orientated norms). “Controlling for” migration rate by including *M* in a multiple regression biases the estimate of conformity’s causal effect on diversity via the path C←U→M←D. Whether it is good or bad to control for any given variable in a regression model, depends on causal assumptions that cannot be found in the data alone. Therefore, all control variables require causal justification (39, 55).

Finally, Fig. 2*D* illustrates how DAGs can be used to represent assumptions about population differences and measurement processes. The selection node S pointing into *A* suggests that populations have different age distributions. Additionally, as DAGs are nonparametric, any time two arrows enter the same node, such as C→D and M→D, either of the two causal pathways might modify (“moderate” or “interact with”) the effect of the other, jointly producing the outcome. This example shows the case where *M* moderates the effect of *C* on *D*; this could mean that conformity has a higher influence on diversity if migration rates are high. Here, researchers need to account for differences in the age structure if they want to generalize or compare findings between populations (see ref. 43 for an introduction to DAGs and poststratification in the context of cross-cultural generalizability). Moreover, until now, we have assumed that we can readily observe all relevant variables. However, researchers are typically not interested in the effects of measured variables per se (e.g., responses in a questionnaire) but in the underlying constructs (e.g., “conformity”) that are assumed to generate the observed choices. Fig. 2*D* illustrates how DAGs can represent measurement processes. Here, three observed measures of conformity (C1–C3) are influenced by the underlying latent variable *C* and by unique unobserved error sources (E1–E3). Latent quantities obtained from factor or item-response-theory models (such as the estimated level of conformity here) are only comparable across populations if—as indicated by the absence of selection nodes here—the same measurement model holds for all populations (see ref. 57 for a guide on DAGs and the comparability of latent constructs in the context of structural equation modeling). To statistically link C1–C3 to *D*, propagating the uncertainty stemming from the measurement process, researchers could simultaneously estimate the latent variable *C* based on observed measures and use that latent score as a predictor of cultural diversity.

At this point, readers might ask how to determine the most appropriate model. Fig. 2 provides a set of credible options, yet numerous alternative model specifications may appear equally plausible, including additional variables such as, for instance, socioeconomic status or education. There are no universal rules for the construction of generative models and the appropriate level of granularity depends on the state of the theory and the specific explanatory goals at hand (see ref. 58 for an argument in favor of simple models). Deriving adjustment sets from all candidate models and comparing linear regressions using AIC, WAIC, or cross-validation might seem intuitive for causal model selection (59). However, these model fit statistics only assess predictive performance, not causal validity, so confounded models may outperform properly specified models (36). A way forward involves assessing models based on their testable implications—statements about relationships that must hold true given the model’s assumptions. Formally, a testable implication asserts either independence between two variables (e.g., M⊥⊥C in Fig. 2*A*) or independence conditional on a set of covariates (M⊥⊥C|A in Fig. 2*B*). A valid model requires the independence condition for all testable implications to be true, so researchers can score all potential candidate models and reject models that fail this critical test. However, there will always be important causal assumptions that cannot be tested with data and many DAGs typically remain empirically consistent with any sample. Theory construction cannot be reduced to empiricism.

### Moving from DAGs to Data.

After selecting an appropriate model (here we assume Fig. 2*B* is the best), we have the basic architecture necessary to formally link theory to data. In our particular case study, we imagine the researcher uses an existing cross-sectional dataset that contains a snapshot of 30 countries from around the world in a single year. For each country, the data have the average age (*A*), a migration rate (*M*), a measure of conformity (*C*), and a measure of cultural diversity (*D*). The researcher can now define the estimand, in this case, the causal effect of migration on cultural diversity or, formally, the difference in the distributions of diversity for a given age structure and level of conformity when migration is set to different values $m_{0}$ and $m_{1}$: $P\left(D|\mathrm{do}\left(M=m_{1}\right),C,A\right)-P\left(D|\mathrm{do}\left(M=m_{0}\right),C,A\right)$ (33).

Using the DAG and the estimand, we can now generate synthetic data. This step is essential for validating the causal identification strategy, and conducting a power analysis. Moving beyond the DAGs to synthetic data requires additional assumptions. Specifically, we need to define the functional relationships between variables, including interactions, and make distributional assumptions about the variables themselves. In Fig. 2*B*, *D* is jointly caused by the migration rate (*M*) and conformity (*C*), but the DAG does not say how they are related to *D*. The researcher must use their ethnographic and scientific knowledge, thinking about interactions and nonlinearity where appropriate. In our case, we consider a simple additive linear relationship and further assume that all variables are standardized. We can now randomly generate 30 data points per variable representing observed values for each country in our dataset (see code repository for details) (28):

[1] A∼ Normal(0,1),

[2] $$M∼\;\text{Normal}(A\beta_{AM},\sigma_{M}),$$

[3] $$C∼\;\text{Normal}(A\beta_{AC},\sigma_{C}),$$

[4] $$D∼\;\text{Normal}(C\beta_{CD}+M\beta_{MD},\;\sigma_{D}),$$

where the *β* weights are parameters that scale the influence of each predictor and the *σ*s controls the amount of unexplained variance due to unmeasured causes. With the ability to generate synthetic data, we can now define a statistical model, which, in this case, is a simple linear regression including *M* and *C* as predictors, closing the confounding path M←A→C→D. Note that the DAG in Fig. 2*B* makes the strong assumption that there is no unmeasured confounding; in reality, populations will often be nonindependent due to spatial proximity and shared cultural ancestry, requiring additional statistical control (43, 60). At this point, we can perform model validation by simulating data with fixed parameter values and ensuring that the statistical model can recover those data-generating values. Additionally, by sweeping across parameters of varying magnitudes and adjusting the amount of unexplained variance, we can learn when our statistical model will begin to fail (see code repository for an example) (28). To infer the implications of potentially important variables we do not know, we could also conduct sensitivity analyses and determine, for example, how strong an unobserved confound would need to be to change a discovered effect (61). Moreover, when different generative models appear plausible, “confusion matrices” can be used to assess how robust our inferences are for alternative model specifications (19).

With a fully validated statistical model, we can compute how adjusting the migration rate will affect the level of cultural diversity for a country with a particular age structure and level of conformity, i.e., a causal effect: in the code repository (28), we simulate and plot the difference in *D* between two levels of *M* (±1SD) marginalizing over different levels of *C* on the outcome scale. In more complex scenarios, the necessary estimator may also be more complex, but it can still be logically derived from a generative model.

### Agent-based Models (ABMs).

DAGs can be extended to represent dynamics in space and time. But when those dynamics as well as the interplay between different levels of analysis (e.g., individual- vs. population-level) are the focus of research, explicitly dynamic generative models with detailed mechanisms complement abstract structural models. Cultural evolutionary research makes frequent use of dynamic population models, and explicitly connecting these to evidence is crucial. For example, the strength of particular demographic forces and the rates of cultural change they create can provide important tests of cultural evolutionary hypotheses (62–68).

**Fig. 3.**
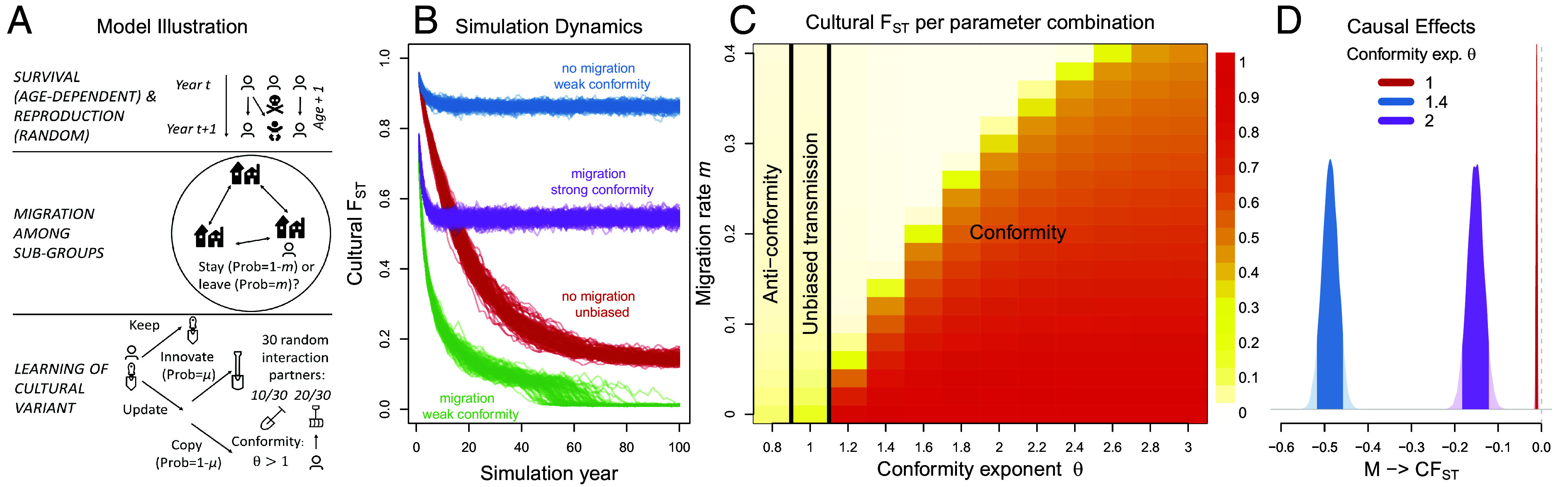
Agent-based model. (*A*) Illustration of one model timestep (see text for detailed description). (*B*) 100 time trajectories of the cultural fixation index ($CF_{\mathrm{ST}}$) for unbiased transmission (*θ* = 1), relatively weak (*θ* = 1.4), and stronger (*θ* = 2) conformity; results are shown for simulations without migration (*m* = 0) and with migration (*m* = 0.2). (*C*) Average $CF_{\mathrm{ST}}$ values for different parameter combinations of conformity exponent *θ* and annual migration rate *m*. Darker colors indicate higher values. Results are averaged over the last 200 time steps of 100 independent simulations per parameter combination. (*D*) Causal effect of migration rate (contrast between *m* = 0.1 and *m* = 0.2) on $CF_{\mathrm{ST}}$ for three different levels of conformity exponent *θ*.

Here, we construct an agent-based model that simulates microlevel processes of birth and death, migration, innovation, and social learning (see Fig. 3*A* for a schematic illustration). This allows us to explore how these forces influence macrolevel patterns of diversity and change. In line with the DAG in Fig. 2*B*, age may influence both migration and learning; implementing these age dependencies in the ABM requires specific functional assumptions that we explain below. Now we model diversity as an emergent property of population dynamics instead of as a direct measurement influenced by named causes. We simulate the evolution of a cultural trait that is characterized by distinct variants (e.g., different languages, beliefs, food preferences, or foraging techniques; represented by different shovels in Fig. 3*A*). These variants produce patterns that can be studied in simulation to understand complex causation in the cultural evolutionary dynamics. The basic causal relationships are the same as before, but now we let the causal implications emerge rather than assuming distributional responses. Agent-based modeling is a major topic of its own (see ref. 30, for a thorough introduction focused on cultural evolution).

#### Model description.

We consider an age-structured population of 3,000 individuals that is divided into 30 equal-sized groups, connected by migration (Fig. 3*A*). Each simulation “year,” individuals survive until next year with an age-dependent survival probability that declines exponentially (at rate *r*_*S*_). Keeping group sizes constant, all adult members of a group have the same probability of producing offspring. Each time step, individuals may migrate to another group either with a fixed probability *m* or with empirically derived age-dependent migration rates (69). Individuals are born naive and must acquire their cultural trait through learning, either individually or socially. Older individuals can also learn but their probability to do so declines (at rate *r*_*L*_) as they age. Learners innovate with probability *μ*, introducing a new variant into the population. With probability 1−μ, they acquire a variant through frequency-dependent social learning. Each social learner randomly selects 30 interaction partners in their local group and adopts variant *i* (of *L* cultural variants held by the interaction partners) with probability proportional to $n_{i}^{\theta }$, where *n*_*i*_ is the frequency of variant *i* among interaction partners and *θ* controls the direction and strength of frequency-dependent bias (23, 50, 70). When *θ* = 1, cultural transmission is unbiased; as *θ* becomes larger than 1, individuals become increasingly likely to adopt high-frequency variants (i.e., conformist). When 0<θ<1, individuals disproportionately adopt low-frequency variants (i.e., anticonformist).

At the end of each timestep, we record the cultural fixation index ($CF_{\mathrm{ST}}$), following the procedure used in ref. 27. Wright’s $F_{\mathrm{ST}}$ describes the proportion of the total variation in (genetic or cultural) variants in a population that occurs between subpopulations rather than within them. When $CF_{\mathrm{ST}}=0$, all cultural variation is due to differences within groups; when $CF_{\mathrm{ST}}=1$, all variation is due to differences between groups. After reaching the demographic equilibrium, we assign unique cultural variants for each group and track how cultural $F_{\mathrm{ST}}$ changes over time (27).

#### Model results.

Starting from maximum between-group diversity, Fig. 3*B* shows how, without conformity, diversity decays even in the absence of migration. While relatively small degrees of conformity (*θ* = 1.4) suffice to maintain diversity between groups if individuals do not migrate, stronger conformity (*θ* = 2) is required to counteract the effect of migration. Fig. 3*C* shows average $CF_{\mathrm{ST}}$ values for a broader range of parameter combinations. In general, between-group diversity is higher if conformity is strong and the migration rate is low. Fig. 3*C* also shows that different combinations of migration and conformity can produce equivalent values of $CF_{\mathrm{ST}}$. For example, unbiased and anticonformist transmission lead to similarly low values of $CF_{\mathrm{ST}}$. Finally, our estimand does not concern cultural diversity per se, but the causal effect of migration on diversity; Fig. 3*D* plots the effect of an increase in migration rate on $CF_{\mathrm{ST}}$ for different levels of conformity. As per the DAG in Fig. 2*B*, these effects are also implicitly conditioning on age, because age is a structural component of the generative model. Overall, increasing the migration rate from *m* = 0.1 to *m* = 0.2 reduces between-group cultural diversity, but it does so at different rates; migration has the strongest effect on $CF_{\mathrm{ST}}$ for an intermediate strength of conformity, a weaker effect for relatively strong conformity, and only negligible effects for unbiased transmission.

#### Longitudinal transmission analysis.

The agent-based simulation tells us how conformity and migration jointly influence between-group diversity in a population with particular demographic and social characteristics. Now suppose we have empirical data on a population. How can we now use data to infer the underlying transmission mechanisms as specified in the model? Our primary estimates are the parameters themselves, i.e., the innovation rate *μ*, conformity exponent *θ*, and the migration rate *m*. These can be used to compute the target estimand, the influence of migration on diversity in the target population. But there is no single parameter for this influence, because diversity emerges from multiple forces at different time scales.

**Fig. 4.**
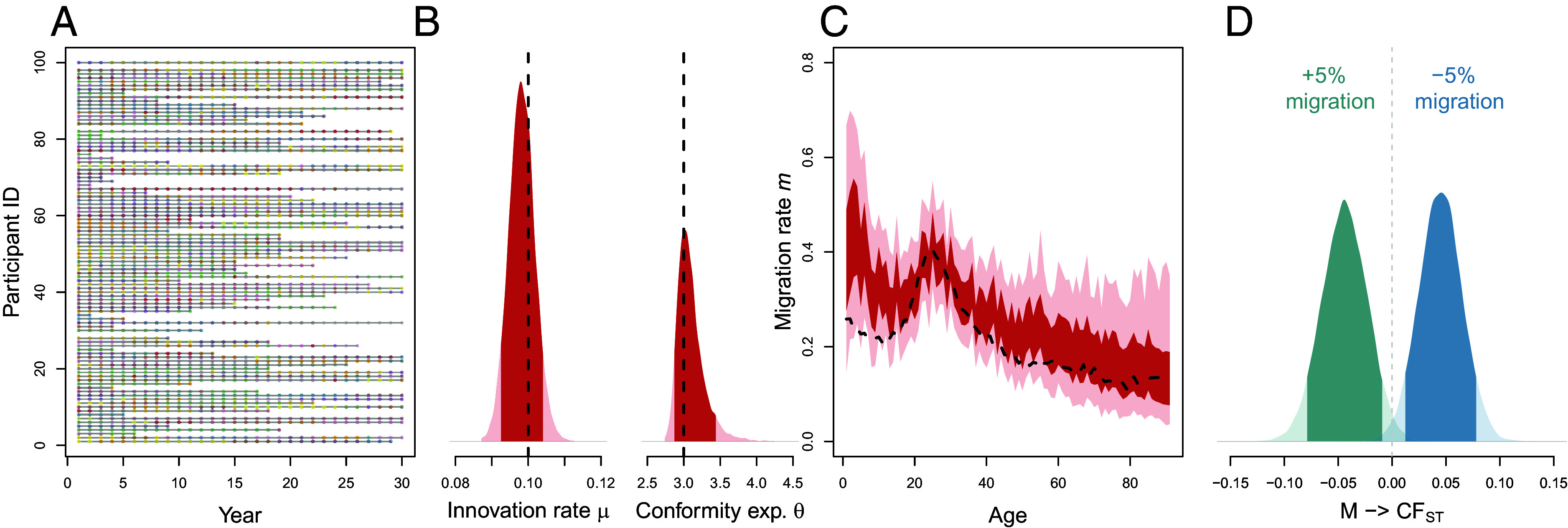
Longitudinal transmission analysis. (*A*) 100 example “participants” (ID on the *y* axis) included in 30 y of simulated data collection; each dot represents data from one participant in 1 y and colors indicate current group membership. (*B*) Full posterior distributions (transparent curves) and 90% highest posterior density intervals (darker curves) for the innovation rate *μ* and the conformity exponent *θ*; the black dashed lines represent the “true” data-generating values. (*C*) Full posterior distributions (transparent areas) and 90% highest posterior density intervals (darker areas) for age-dependent migration rates; the black dashed line represents the underlying age trajectory taken from ref. 69. (*D*) Causal effect of migration rate on $CF_{\mathrm{ST}}$ (±5% migration in each age class compared to “observed” rates).

We first consider an enviable research case: researchers have collected individual-level data on a cultural trait over a 30-y period (Fig. 4*A*). Longitudinal research that tracks the same individuals over time is most useful. But when the data are less than optimal—the usual situation—the generative model logically constrains which inferences we can justify. In a later section, we expand on this point. Our simulated researchers not only recorded the cultural variant of each individual in a given year as well as their group membership but also each individual’s most important social contacts as well as the variants of those contacts. In the first wave of data collection, 500 individuals were included in the dataset and 179 remained in the population until the final year. This dataset is generated under known parameter values, and thus the analytic procedure outlined here also constitutes a basic validation test that should be an ordinary part of a computational workflow.

With fine-grained time-series data on individuals’ cultural variants as well as their social networks, we can directly use our generative agent-based model as a statistical model (36). For each year *t*, we first model whether individual *j* innovates a new variant, $I_{j,t}∼\;\text{Bernoulli}\left(\mu_{j}\right)$, and whether they migrate to another group, $M_{j,t}∼\;\text{Bernoulli}\left(m_{a[j,t]}\right)$, where $I_{j,t}$ and $M_{j,t}$ represent 0/1 variables (hence, the Bernoulli likelihoods) and the subscript *a* indicates that we assumed different migration rates for different ages. In case individual *j* did not innovate, we further model their probability to adopt a given variant (held by themselves or their *L* contact in year *t*), $C_{j,t}∼\;\text{Categorical}\left(p_{j,t}\right)$, where $p_{i,j,t}$ for variant *i* is—identically to the ABM—proportional to $n_{i,j,t}^{\theta_{j}}$. We use a categorical likelihood function because the cultural variant an individual adopts is a discrete variable with more than two possible outcomes.

Using the Stan probabilistic programming language (71), we sample from the joint posterior distribution of *μ*, *θ* (Fig. 4*B*) and age-specific migration rates *m* (Fig. 4*C*). Once we have estimates, we can use the agent-based model again and simulate cultural dynamics for the whole population and at longer timescales, exploring a variety of counterfactual scenarios and generalizations. As an example, Fig. 4*D* shows the causal effect of increasing or decreasing age-dependent migration rates by 5%, marginalizing over the uncertainty in both *μ* and *θ*. This change in migration causes symmetrical shifts in $CF_{\mathrm{ST}}$ in the direction expected from Fig. 3*C*.

#### Approximate Bayesian computation (ABC).

Long-term individual-level data are ideal for inferring cultural evolutionary processes. But in many observational and also experimental scenarios, we do not have access to detailed time-series of transmission events, preventing the use of fully likelihood-based inference. In such scenarios, likelihood-free approaches like ABC can be used to sample from the joint posterior distribution of parameters (40). ABC depends on a detailed generative model which acts as the inferential model, as it is directly used to produce data which is compared to data collected in a real-world population. This allows us to see which inferences can be justified when the time and spatial scales of our generative theory differ from those of the available evidence. ABC has seen successful application in cultural evolution settings and is most widespread in archaeological applications where the data nearly always lack the resolution of the generative model (40, 72–76). ABC is increasingly used in other areas of cultural evolutionary research (77–80).

We formulate a simple ABC analysis, to address the same goals as in the longitudinal time-series, but without access to longitudinal data. We use the agent-based model to generate a cross-sectional dataset that represents data that would be collected in a more typical research context. We then compare this single dataset, as if it were actual data, to simulated datasets generated from the ABM, varying the generative parameters (m=[0,0.4],θ=0.5,1,2,3) across the parameter space explored in this manuscript. The ABC algorithm calculates the difference between the reference and simulated data, based on the $CF_{\mathrm{ST}}$ value, using a rejection algorithm to quantify the combinations of parameter values that can generate datasets similar to the reference data (40). We take 1,000 samples from the joint posterior, which denote parameter combinations, that when input into the ABM, produced the smallest difference between the generated $CF_{\mathrm{ST}}$ and the reference $CF_{\mathrm{ST}}$ that stood in for real-world data.

We present an ABC analysis conducted with two reference datasets: d_unbiased, generated with unbiased transmission (*θ* = 1) and a migration rate of *m* = 0.3, and d_conformist, generated with conformist transmission, (*θ* = 2), and a migration rate of *m* = 0.1. In each case, the reference dataset is compared to 100,000 datasets simulated from the generative model under varying parameter values.

**Fig. 5.**
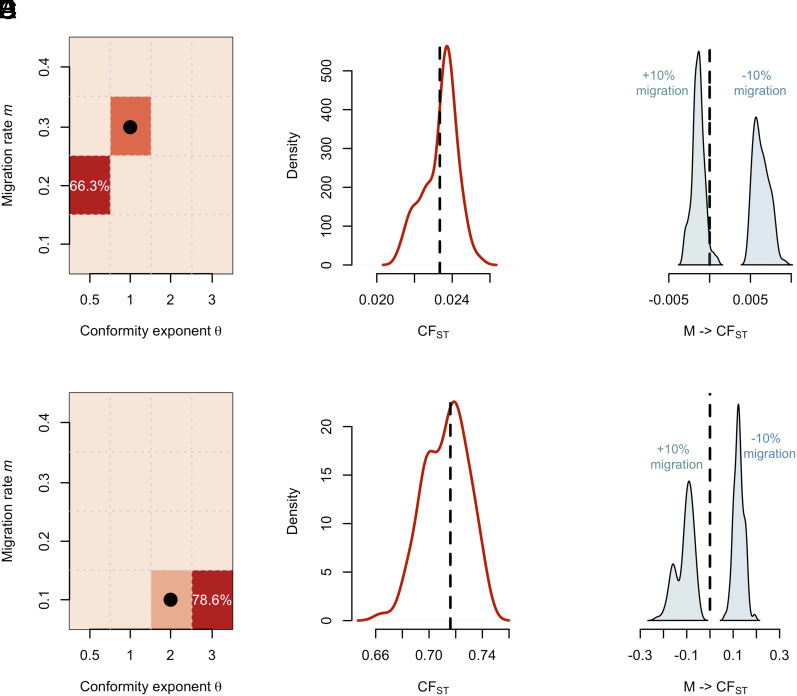
ABC results. The first row shows the results of an ABC analysis conducted for a reference dataset generated with input values *θ* = 1 for the conformity exponent, and *m* = 0.3 for the migration rate (d_unbiased). Panel (*A*) shows the joint posterior of the conformity exponent *θ* and migration rate *m*. The intensity of red indicates higher frequencies of particular parameter combinations in the joint posterior, i.e., how many of the 1,000 samples represent a particular combination of migration rate and conformity exponent values. The black circle indicates true values used to generate the reference data. Panel (*B*) shows the posterior prediction of $CF_{\mathrm{ST}}$ for 100 datasets generated from the joint posterior. The black dashed line represents the reference value. Panel (*C*) shows the effect of a ±10% change in migration rate on $CF_{\mathrm{ST}}$, as implied by the joint posterior. The second row presents the results of an ABC analysis for reference_data with input values *θ* = 2, and *m* = 0.1 (d_conformist). Panel (*D*) presents the joint posterior of conformity exponent *θ* and migration rate *m*. Panel (*E*) shows the posterior prediction of $CF_{\mathrm{ST}}$ for 100 datasets generated from the joint posterior, while Panel (*F*) demonstrates the effect of a ±10% change in migration rate on $CF_{\mathrm{ST}}$.

For d_unbiased (Fig. 5, top row), the joint posterior indicates the reference data were most likely produced with values of *θ* = 0.5 and *m* = 0.2 (66.3% of posterior), with 33.7% of the posterior accounting for the true values *θ* = 1 and *m* = 0.3. The model is certain the data were not generated under conformist transmission, but cannot reliably distinguish anticonformity and unbiased transmission. Fig. 3*C* shows that the $CF_{\mathrm{ST}}$ values associated with unbiased and anticonformist transmission as well as with migration rates of *m* = 0.2 and *m* = 0.3 are broadly the same. Further illustrating this equifinality, the $CF_{\mathrm{ST}}$ values generated from the posterior map well onto the reference $CF_{\mathrm{ST}}$ from d_unbiased (Fig. 5*B*). Thus, the model can predict $CF_{\mathrm{ST}}$ values accurately but cannot recover the true parameters that generated them.

For d_conformist (Fig. 5, bottom row), a similar pattern emerges: while the posterior strongly indicates conformist transmission (no posterior samples for *θ* ≤ 1), and correctly identifies the migration rate (*m* = 0.1), it misses the true value of *θ*. This uncertainty stems from the fact that $CF_{\mathrm{ST}}$ values generated by *m* = 0.1 and *θ* = 2 cannot be reliably distinguished from values generated by *m* = 0.1 and *θ* = 3 (Fig. 3*C*). Again, while the model cannot identify the true generative values, the posterior prediction accurately captures the $CF_{\mathrm{ST}}$ value from d_conformist (Fig. 5*E*). One major advantage of generative inference is that the equivalence between the generative and statistical model allows us to better understand uncertainty in the posterior, and sources of equifinality. Under this particular generative model, a cross-sectional dataset would not be enough to correctly estimate the conformity exponent, and depending on the exact nature of the reference data, could also struggle with the migration rate. However, qualitatively, the model is able to identify the transmission process.

As before, once we have estimates, we can infer effects or make predictions for a target population, by drawing from the joint posterior and pushing parameter estimates through the generative model. This step is the same as in the ideal-data case, the longitudinal analysis, but the additional uncertainty in estimates will project through to uncertainty in causal effects. As an example, we calculate the effect of increasing and decreasing migration rate by 10%. For both datasets, increasing migration rate by 10% decreases between-group cultural diversity, and vice versa for decreasing migration rate. Echoing the results in Fig. 3*D*, the effect of migration is stronger with conformist compared to unbiased transmission. These results highlight the value of being able to interpret statistical analyses with theoretical model predictions in hand.

ABC is an active area of statistical research. We present a simple rejection algorithm, but ABC is a growing field and researchers should familiarize themselves with the expanding possibilities (81, 82). Moreover, there are now also similar likelihood-free inference approaches based on artificial neural networks (83). Generative inference opens the doors to concrete mechanistic modeling with imperfect data, but it also requires a refocus of analytic methodologies, toward greater engagement with generative models, and a strong commitment to analysis pipeline validation.

## Conclusion

Widespread nonreproducibility (84, 85), publication bias (86), and the failure of prominent findings in biology and the behavioral sciences to replicate (87), show that powerful statistical methods are not enough to produce reliable research. The way that statistical analysis is incorporated into research is too often tangled and unjustified. Modeling decisions are poorly justified, and connections to scientific goals are too often metaphorical rather than logical. There has been important emphasis on initiatives like preregistration (88) and registered reports (89). But there has been much less on the knowledge and tools needed to develop and justify analysis plans and their contingencies. It is too easy for false results to replicate and appear superficially reliable (90). Statistical methods should be integrated into research in a way that allows them to reliably and transparently address scientific goals.

In pursuit of this integration, we have illustrated a scientific workflow for cultural evolution (and beyond) that allows researchers to bridge between theory and data in a principled and transparent way. Based on generative models of empirical phenomena that can take various forms depending on the knowledge we have and the assumptions we are willing to make, our workflow outlines how to clearly define an inferential target and how to construct inferential models to estimate it.

While highlighting the central role of theory and computational methods, the approach also invites ethnography and empirical domain knowledge to bear on analytic procedures. Ethnographic context should inform every aspect of a scientific project—from conception, operationalization, methodology, to analysis—to ground cultural evolution in the specific sociodemographic processes of the past and present peoples we seek to understand (91). Generative inference is only as good as the generative models underlying it. These models depend on unambiguous and well-asserted assumptions that build on both theoretical and ethnographic insights, allowing for greater integration with the detailed knowledge researchers often have about the populations they work with. For example, the inference of learning mechanisms in our example workflow crucially depends on assumptions about the number of social interaction partners relevant to the transmission of the cultural trait under study. Some traits might only be adopted from close kin, whereas others might potentially be learned from any person people interact with. These assumptions can only reliably be made based on detailed observations in the community as well as people’s own reports about possible transmission pathways (92, 93). Likewise, we have detailed various ways to use posterior estimates for further inference about causal effects, (out-of-sample) predictions, and counterfactuals, all dependent on well-defined real-world explanatory goals and target populations. Therefore, to understand cultural evolution in situ, we call for the development of more tailored “mid-level” theory and bespoke computational workflows, that translate between abstract theory, on the one hand, and situated ethnographic knowledge on the other. This also facilitates entering into a dialog with study participants, collaboratively investigating which assumptions and processes might best capture cultural transmission in their community.

For the purposes of clear demonstration, we have chosen a straightforward example, perhaps deceptively so. For researchers who have clear theoretical expectations and collect data which matches the structure required by their generative model, the workflow presented here is comprehensive. Beyond such desirable situations, generative models can be extended to deal with various imperfections of the real world, such as measurement issues, uncertainty in the state or observation process, missingness, or imbalanced sampling (36). The core strategy is always to build probabilistic models of the processes that produced the data at hand—be they scientifically meaningful or accidental. That is, data missingness and selection effects can, and should be, explicitly modeled, so that we may understand their effects on inference and better account for them. Moreover, utilizing a generative framework can help clarify exactly what sort of data are required for reliable inference and which assumptions need further testing, facilitating research design and sample collection. For example, certain areas of the parameter space of the generative model may produce maximally distinctive patterns, suggesting data from these areas to be most productive.

Fieldwork can also be combined with controlled experiments to explicitly test and refine key assumptions of the generative model. By investigating the effect of certain variables while keeping others constant, experiments may also serve as an important bridge between abstract models and the real world. Importantly, even randomized experiments need careful reasoning about the inferential workflow and causal assumptions (94). Conditioning on posttreatment variables by, for example, dropping participants based on failed manipulation or attention checks or including downstream mediators in a model, can bias estimates of treatment effects (95). Moreover, without a logically derived estimand, differences in within-group comparisons (e.g., changes over time) are often falsely interpreted as evidence for treatment effects; different effects do not imply an effect on the differences (96).

We end by re-emphasizing the iterative nature of the proposed workflow. Rare is the research project that starts with enough information to execute each of the proposed steps perfectly. Instead, looping through the workflow should be seen as a process of iterative learning, about the generative process, the people we work with, and the data limits and potentials.

## Data Availability

Annotated scripts for all workflow examples have been deposited on GitHub (https://github.com/DominikDeffner/CulturalEvolutionWorkflow) (28).
